# Assessment of the Relation Between Patterns of Third-Molar Impaction and Blood Group: A Retrospective Study

**DOI:** 10.7759/cureus.45130

**Published:** 2023-09-12

**Authors:** Muath S Alassaf, Saad M Hasubah, Shadan H Sharbib, Ahmad A Othman, Mahmoud A Alsulaimani, Ahmad A Qazali, Marwan A Alqurashi, Ahmed S Khoshhal

**Affiliations:** 1 Department of Orthodontics and Dentofacial Orthopedics, Taibah University, Madina, SAU; 2 Department of Dental Education, Taibah University, Madina, SAU; 3 Department of Oral and Maxillofacial Surgery, Taibah University, Madina, SAU; 4 Department of Prosthodontics, Taibah University, Madina, SAU; 5 Department of Restorative Dentistry, Dental Center in Ohud Hospital, Madina, SAU

**Keywords:** abo blood-group system, rh factor, impacted tooth, blood groups, third molar

## Abstract

Background

The prevalence of impacted third molars is high in the global general population. The etiology of impacted third molars is not fully understood, but it is thought to result from combined genetic and environmental factors. Some studies have suggested a link between the blood group and the risk of impacted third molars. This study aimed to investigate the association between the blood group and the presence of impacted third molars and its pattern.

Method

A total of 856 panoramic radiographs were included in the study. The third molars were evaluated for the pattern of third-molar impaction and blood characteristics recorded as ABO group and presence or absence of Rhesus antigen.

Results

The results showed no significant association between the blood group and the presence of impacted third molars. The prevalence of at least one impacted the third molar was 34.6%. The most common angulation of impacted third molars was vertical (V) (45.1%), followed by mesioangular (MA) (33.7%), distoangular (DA) (13.8%), and horizontal (H) (7.4%). There was no significant association between the blood group and the number of impacted third molars nor between the blood group and the angulation of the impacted third molars.

Conclusion

This study suggests that the blood group is not a major factor in the development of impacted third molars. However, further studies with larger sample sizes are needed to confirm these findings.

## Introduction

Third-molar teeth are present in most adults and generally become apparent between the ages of 18 and 24 years with wide variation in the age of presentation [1]. Impacted third molars are not usually expected to erupt into functional occlusion. Third molars become partially or completely impacted by lack of space, obstruction, or abnormal position. Impacted third molar may be diagnosed through symptoms such as pressure, pain, or swelling and physical examination with probing or direct visualization; or incidentally by routine dental radiography. Radiographic examination of the third molar is important in estimating the age of individuals and in treatment planning [2].

Multiple classifications were proposed for impacted third molars. Based on the angulation of the third molar in relation to the second molar impactions may be classified as vertical (V), horizontal (H), mesioangular (MA), and distoangular (DA). The etiology of the high rate of impacted wisdom teeth has not yet been stated clearly, but common findings are lack of jaw space, presence of pathology, and malposition of adjacent tooth [3,4].

In most cases, management of impacted third molars consists of extraction, although the molar can be kept if it is not symptomatic and does not have a pathologic lesion. Multiple possible complications may arise from an impacted third molar, such as the development of cystic lesions, caries in the adjacent second molar, and pericoronitis. Prophylactic removal of third molars is preferably done before age 25 [5,6].

Blood types are an inherited trait, but their distribution varies within cultures and races. The two categorization systems most frequently used are ABO classification and the Rhesus (Rh) system [7]. ABO classification is based on the presence of anti-A or anti-B antibodies in the serum, as well as A and B antigens on the surface of red blood cells. Antigen A is present in blood type A red blood cells, and anti-B antibodies are present in serum. Antigen B and anti-A antibodies are found in blood type B. A and B antigens are present in blood type AB; however, there are no antibodies. Blood type O has both anti-A and anti-B antibodies but no antigens [8].

Along with A and B antigens, red blood cells also have the Rh factor on their surface. Rh-positive individuals have the Rh factor, whereas Rh-negative individuals do not. Limited research was carried out on the association between blood types and oral disease, resulting in positive findings. Previous studies focused on the association between blood types and medical and dental conditions such as carcinomas, gastric ulcers, and hypertension. In the dental field, studies on malocclusion, periodontal disease, dental caries, and salivary gland tumors were done [9-11]. Some studies, however, mentioned the association of ABO and Rh blood groups and impacted teeth in different populations, including Malaysian, Iranian, and Indian populations. All three previous studies found no association between the blood groups and pattern of impacted third molars; however, all had studied the relation on a relatively small sample size [12-14].

To determine which group of individuals is more likely to develop impacted third molars and to better target interventions, it is crucial to identify the relationship between ABO and Rh blood groups and impacted teeth.

This study aims to investigate any relation between the blood group and the behavior of third molars.

## Materials and methods

Population

A cross-sectional study included 1500 patients who came to Taibah University Dental Clinics seeking treatment between January 2022 and January 2023. All patients who obtained panoramic radiographs are considered part of the study population.

Inclusion criteria were set to include Saudi patients with ages above 18 years and good orthopantomography (OPG) quality clearly showing the areas of third molars. All cases with pathology related to third molars or its area such as cysts, expansile lesions, or severe bone loss were excluded. Also, patients with a history of blood disorders or congenital syndromes were excluded. Included patients were then contacted to obtain their blood groups as registered by the Ministry of Health.

All OPGs were screened for quality assessment and filtered to keep those that met the inclusion criteria. Individuals who voluntarily agreed to participate in the study were included.

Design

**Figure 1:**
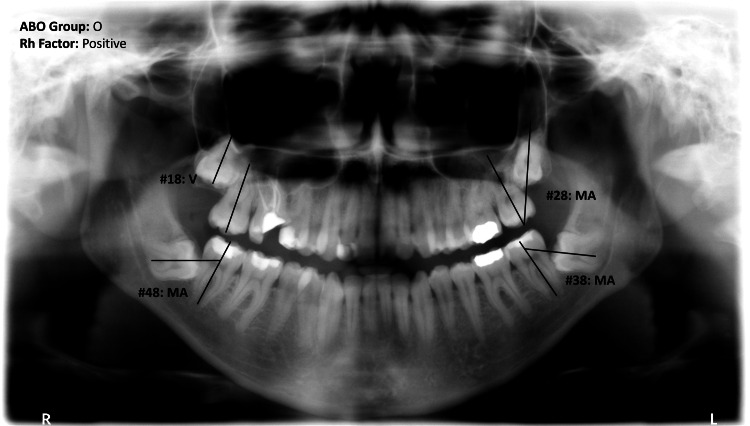
Orthopantomograph of the 25-year-old patient showing the classification of impacted third molars with the blood group MA, mesioangular; V, vertical

Two investigators screened the existing radiographs (1500), contacted the patients who met the inclusion criteria (856) to obtain the blood group as registered in the patient's health card by the Ministry of Health, and checked for any significant medical history. The data was collected in a spreadsheet containing the following variables: gender, age, and third-molar status (not present, erupted, impacted). Impacted third molars were classified based on angulation MA, DA, V, or, V. The blood group was recorded (A, B, AB, or O), and the Rh factor was recorded as positive or negative. Figure 1 shows an OPG with the third molars classification and blood group.

Ethical considerations

The study idea and method were explained to all patients, and a waiver of the written consent form was approved by the Research Ethics Committee at Taibah University at the College of Dentistry. Ethical approval Number (TUCDREC/140623/MSAlassaf)

Statistical analysis

Data were coded and analysis was done using Statistical Package for the Social Sciences (SPSS version 23). A descriptive analysis of the variables as percentage and frequency for qualitative data and mean and standard deviation for qualitative variables was conducted. Then, in the comparison process between groups, chi-squared tests were used with a significance level set at a p-value of ≤0.05.

## Results

Of the screened (1500) panoramic radiographs, 856 were included in this study. Male subjects accounted for 739 (86.33%) of the cases. Age ranged from 18 to 85 with a mean age of 34.78 (±13.68), with 51.4% between 21 and 30 years. Table 1 summarizes the age groups of the population (n=856).

**Table 1:** Age groups of the studied population with at least one impacted third molar (n=296)

Age	Count	%
Less than 21	62	20.9
21 to 30	152	51.4
31 to 40	45	15.2
41 to 50	29	9.8
51 to 60	6	2.0
61 to 70	2	0.7

Among these cases 560 (65.4%), OPGs showed no impacted third molars. The pattern of impaction in the remaining 296 (34.6%) is shown in Table 2.

**Table 2:** Pattern of impaction per third molar (n=672)

Classification of third molar	#18	#28	#38	#48	Total
Mesioangular	27(19.42%)	28(19.17%)	97(50.52%)	89(45.64%)	241(134.7%)
Distoangular	30(21.58%)	34(23.28%)	10(5.20%)	10(5.12%)	84(55.2%)
Horizontal	0(0%)	2(1.36%)	22(11.45%)	33(16.92%)	57(29.75%)
Vertical	82(58.99%)	82(56.16%)	63(32%)	63(32.3%)	290(180.2%)
Total	139(100%)	146(100%)	192(100%)	195(100%)	672(400%)

Impacted third molars in the mandible presented 285 (42.41%) and in the maxilla 387 (57.59%). In addition, the right side presented 334 (49.7%) cases of impacted third molars, while the left side accounted for 338 (50.3%). There was no statistical difference between the maxilla and mandible or between the right and left sides (p>0.05). 

**Figure 2:**
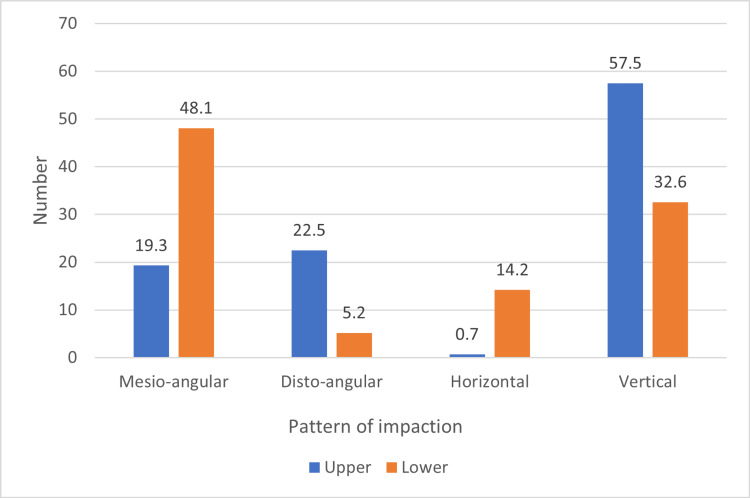
Distribution of impacted third molars in the maxilla and mandible (n=672)

Regarding the impaction pattern, in the maxilla, the most common was V (57.5%), and the least common was H, accounting only for two cases (0.7%). In the mandible, the most common was MA (48.1%), and the least common was DA (5.2%). Figure 2 shows the pattern of impaction per jaw.

**Figure 3:**
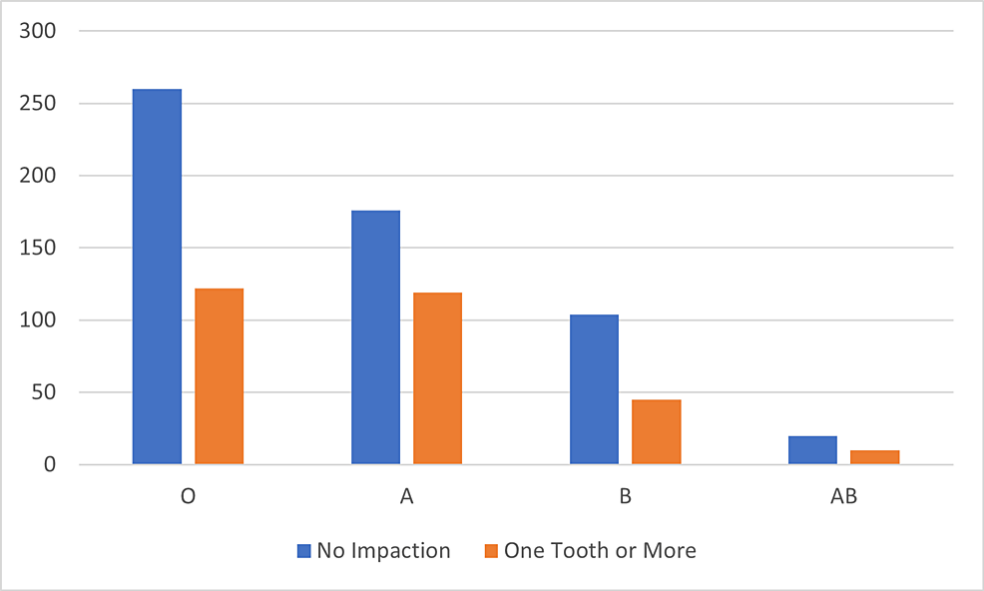
Distribution of blood groups with the presence or absence of impacted third molar

The prevalence of the presence of at least one impacted third molar was 34.5%. In the studied sample, the blood group O was the most common (44.60%), followed by A, accounting for 34.50%; then B and AB, comprising 17.40% and 3.50%, respectively. For the Rh factor, only 7.4% were negative. The distribution of cases with at least one impacted third molar and ABO blood groups is shown in Figure 3.

Table 3 and Table 4 show the third molar status in relation to the ABO grouping system and the Rh factor, respectively. The chi-square test showed no significant relation to ABO grouping or Rh factor and the number of impacted third molars.

**Table 3:** Third molars' status in relation to ABO blood grouping system (n=856)

Number of impacted teeth		ABO group					
		O	A	B	AB	Total	P-value
No impaction	Count	260	176	104	20	560	>0.05
	% of total	30.40%	20.60%	12.10%	2.30%	65.40%	
One tooth	Count	36	40	17	2	95	
	% of total	4.20%	4.70%	2.00%	0.20%	11.10%	
Two teeth	Count	40	38	11	3	92	
	% of total	4.70%	4.40%	1.30%	0.40%	10.70%	
Three teeth	Count	17	17	7	2	43	
	% of total	2.00%	2.00%	0.80%	0.20%	5.00%	
Four teeth	Count	29	24	10	3	66	
	% of total	3.40%	2.80%	1.20%	0.40%	7.70%	
Total	Count	382	295	149	30	856	
	% of total	44.60%	34.50%	17.40%	3.50%	100.00%	

**Table 4:** Third molars' status in relation to Rh factor (n=856)

Number of impacted teeth		Rh factor		Total	P-value
		Positive	Negative		
No impaction	Count	505	55	560	>0.05
	% of total	59.0%	6.4%	65.4%	
One tooth	Count	88	7	95	
	% of total	10.3%	0.8%	11.1%	
Two teeth	Count	86	6	92	
	% of total	10.0%	0.7%	10.7%	
Three teeth	Count	40	3	43	
	% of total	4.7%	0.4%	5.0%	
Four teeth	Count	60	6	66	
	% of total	7.0%	0.7%	7.7%	
Total	Count	779	77	856	
	% of total	91.0%	9.0%	100.0%	

Table 5 shows the distribution of occurrence of different angulations per third molar in relation to ABO blood groups. No statistically significant association was found among the blood groups.

**Table 5:** Status of the third molars in relation to ABO blood groups among the studied population (n=856)

#18 in relation to the ABO grouping system						
Blood group	Erupted	Not present	Mesioangular	Distoangular	Horizontal	Vertical
O	18.20%	19.50%	1.40%	1.80%	0.00%	3.70%
A	12.40%	16.20%	1.40%	0.80%	0.00%	3.60%
B	6.70%	7.90%	0.40%	0.60%	0.00%	1.90%
AB	1.30%	1.50%	0.00%	0.40%	0.00%	0.40%
Total	38.60%	45.20%	3.20%	3.50%	0.00%	9.60%
#28 in relation to the ABO grouping system						
Blood group	Erupted	Not present	Mesioangular	Distoangular	Horizontal	Vertical
O	17.90%	19.60%	1.60%	2.00%	0.10%	3.40%
A	13.10%	15.00%	1.20%	1.30%	0.10%	3.90%
B	8.20%	6.50%	0.40%	0.50%	0.00%	1.90%
AB	0.90%	1.80%	0.10%	0.20%	0.00%	0.50%
Total	40.10%	42.90%	3.30%	4.00%	0.20%	9.60%
#38 in relation to the ABO grouping system						
Blood group	Erupted	Not present	Mesioangular	Distoangular	Horizontal	Vertical
O	16.50%	18.60%	4.30%	0.70%	1.20%	3.40%
A	11.70%	13.80%	5.50%	0.20%	0.90%	2.30%
B	6.90%	7.40%	1.10%	0.20%	0.40%	1.50%
AB	0.90%	1.90%	0.50%	0.00%	0.10%	0.10%
Total	36.00%	41.60%	11.30%	1.20%	2.60%	7.40%
#48 in relation to the ABO grouping system						
Blood group	Erupted	Not present	Mesioangular	Distoangular	Horizontal	Vertical
O	18.60%	16.60%	3.70%	0.50%	1.90%	3.40%
A	11.80%	13.20%	5.00%	0.50%	1.40%	2.60%
B	6.20%	8.20%	1.20%	0.20%	0.60%	1.10%
AB	1.60%	1.10%	0.50%	0.00%	0.00%	0.40%
Total	38.20%	39.00%	10.40%	1.20%	3.90%	7.40%

## Discussion

The most commonly impacted teeth in the mouth are the third molars. In previous studies in Saudi Arabia, the prevalence of at least one impacted third molar ranged from 12.31% to 40.5% [15,16]. In our study, 34.6% of the 856 included cases, or about one-third of the population, has one impacted third molar or more. This number is similar to the reported prevalence in studies conducted in Saudi Arabia and other countries [15]. However, it is lower than the reported prevalence in Turkey (47.5%) or Oman (54.3%) [17,18]. The variation in calculated prevalence is likely due to differences in the sampled population and sampling methods, but as far as this study suggests, this is not related to blood groups.

Agewise, previous studies mentioned that the most common decades for diagnosis of impacted third molar are the third and fourth but, in our study, about half of patients were between the ages of 20 and 30 years, and some studies were consistent with this finding [18,19].

These inconsistencies in age are mostly related to the variability in the sample and the attitude toward prophylactic extraction of the third molars. The third molar is usually removed before the age of 25 [5].

In the mandible, the pattern of impaction was mostly MA, V, H, and least DA, which is consistent with multiple studies worldwide and in Saudi Arabia. For the maxilla, the order is V, DA, MA, and rarely H, which is also similar to the findings of other studies [19]. This repeating pattern of impaction is likely due to the growth model of the jaws, as stated in different studies. The lack of jaw space is the most common cause of impacted third molars and is related to genetics. So, a relationship between blood group and third-molar impaction is plausible [20].

Multiple studies have demonstrated links between diseases or conditions and blood groups, given that certain blood groups had higher or lower risks for a condition than others. For example, blood group A has been linked to multiple carcinomas, including salivary glands, cervix, uterus, bladder, and rectum, in addition to pernicious anemia. Blood group O has been linked to gastric ulcers, rheumatoid arthritis, Von Willebrand disease, and typhoid. *Escherichia coli*, gonorrhea, and urinary tract infections are associated with group B. A study investigating the relationship between hypertension and blood group after menopause found a significantly higher risk of hypertension for post-menopausal women [10,21]. Recently, a study by Jiao Zhao et al. investigating the risk of COVID-19 for ABO groups concluded that people with blood group A were more at risk for COVID-19 than people with blood group O [22].

Studies related to conditions affecting the oral and maxillofacial region included more clinical attachment loss in periodontal tissues with blood group B [23]. This could be related to the presence of higher percentages of lymphocytes in the white blood cell count in patients with group B [21,24]. Moreover, blood group O had a significantly lower risk of periodontitis [25]. Another study found that denture stomatitis is more common in blood group O [26]. What might explain these findings is that the conditions found to be related to blood groups are involved in immunological factors whether they are inflammatory or oncology-related conditions.

In the aspect of dentofacial morphology and malocclusion, no statistical difference was found by Shokor et al., which evaluated 200 Malaysian patients, and Gupta et al., which evaluated 385 patients in Nepal. Also, studies done in Iraq showed no relation between malocclusion and blood groups [27].

In contrast, Sharma et al. evaluated 300 Indian patients and showed that malocclusion was highest in B, A, and O, with less in AB, with statistical significance [28]. Rashid studied 300 Egyptian patients and found the statistical difference, with A as the most common, then O, followed by B, and finally AB [29]. A study by Gheisari et al. evaluating maxillofacial deformities also showed an association with blood groups, in which group B, then AB, had an increased incidence of association with maxillofacial deformities [16]. As the pattern of malocclusion differs in different populations, the status of third molars may also differ. 

The relationship between blood groups and the impaction of third molars has been evaluated in a few studies. The first, which was conducted in 2019 by Zhi YH et al. and included 241 patients from orthodontic clinics in Malaysia, found no significant association, but the highest impacted third molars were in A, O, and AB, and the least in B without evaluation of Rh factor [14]. Then, in 2016, the study of Narang D et al. on 250 patients with impacted third molars also found no relation, with the highest rate occurring in AB, followed by O, A, and B [12]. The most recent study in 2021 by Ahmadi et al., examining 115 cases with impacted third molars, found that the rates of impaction in B and AB groups were higher than those in O and A groups, in contrast to our study, in which O and A accounted for most cases. Both studies were consistent regarding the Rh factor [13]. Among these studies, the pattern of impaction was evaluated by Zhi YH et al. only, compared with our study, for #18, the highest pattern was vertical in group A, whereas in our study it is vertical in group O; #28 and #38 had their highest pattern as MA also with group A, whereas in our study it is vertical with group A for #28 and MA with group A for #38, which is the same as in the Zhi YH et al. study. In the Zhi YH et al. study, #48 was MA in group B, whereas group A was MA in our study. Only one study, that of Al-Molla et al., was related to the role of blood group in the eruption of third molars, which revealed no association, as in our study [30].

Limitation

The study focuses on the association between blood groups and the pattern of impacted third molars in Saudi Arabia. However, the study is limited by a predominantly male sample. Even though the sample size is larger than the previous studies, different findings might emerge with an even larger sample. Further analysis could be beneficial for examining the pattern of impacted third molars and malocclusion in the same study.

## Conclusions

This study found that nearly one-third of Saudis have at least one impacted wisdom tooth. Impaction is more common in people between the ages of 20 and 30. Based on the data of this study, there is no association between blood type and wisdom tooth impaction. However, the study was limited in diversity, so more research is needed to confirm these findings.
